# Disaggregation and invasion of ovarian carcinoma ascites spheroids

**DOI:** 10.1186/1479-5876-4-6

**Published:** 2006-01-24

**Authors:** Kathryn M Burleson, Matthew P Boente, Stefan E Pambuccian, Amy PN Skubitz

**Affiliations:** 1 Department of Laboratory Medicine and Pathology, University of Minnesota Medical School, University of Minnesota, Minneapolis, Minnesota, USA; 2 Department of Obstetrics, Gynecology and Women's Health, Division of Gynecologic Oncology^†^, University of Minnesota, Minneapolis, Minnesota, USA

## Abstract

**Background:**

Malignant ascites often develops in advanced stages of ovarian carcinoma, consisting of single and aggregated tumor cells, or spheroids. Spheroids have commonly been used as tumor models to study drug efficacy, and have shown resistance to some chemotherapies and radiation. However, little is known about the adhesive or invasive capabilities of spheroids, and whether this particular cellular component of the ascites can contribute to dissemination of ovarian cancer. Here, we examined the invasive ability of ascites spheroids recovered from seven ovarian carcinoma patients and one primary peritoneal carcinoma (PPC) patient.

**Methods:**

Ascites spheroids were isolated from patients, purified, and immunohistochemical analyses were performed by a pathologist to confirm diagnosis. *In vitro *assays were designed to quantify spheroid disaggregation on a variety of extracellular matrices and dissemination on and invasion into normal human mesothelial cell monolayers. Cell proliferation and viability were determined in each assay, and statistical significance demonstrated by the student's t-test.

**Results:**

Spheroids from all of the patients' ascites samples disaggregated on extracellular matrix components, with the PPC spheroids capable of complete disaggregation on type I collagen. Additionally, all of the ascites spheroid samples adhered to and disaggregated on live human mesothelial cell monolayers, typically without invading them. However, the PPC ascites spheroids and one ovarian carcinoma ascites spheroid sample occasionally formed invasive foci in the mesothelial cell monolayers, suggestive of a more invasive phenotype.

**Conclusion:**

We present here *in vitro *assays using ascites spheroids that imitate the spread of ovarian cancer *in vivo*. Our results suggest that systematic studies of the ascites cellular content are necessary to understand the biology of ovarian carcinoma.

## Background

Ovarian carcinoma is the most lethal cancer of the female reproductive system. Despite decades of research, the long-term survival rate for the disease is poor, with only 25% of patients diagnosed with stage III or IV ovarian cancer surviving beyond five years. Typically, 75% of patients have advanced stage disease at the time of initial diagnosis, due to an absence of symptoms and the lack of a reliable detection method in early stages of the disease [1].

Ovarian cancer spreads as tumor cells are shed from the surface of the ovary into the peritoneal cavity. The tumor cells can secrete vascular permeability factors and block lymphatic drainage, leading to the accumulation of ascites fluid [2-5]. Ovarian tumor cells sloughed into the ascites can use the fluid as a means to seed the peritoneal cavity, adhering to the mesothelial cells that line the peritoneum and its organs. Once adhered, the tumor cells can subsequently invade, establishing tumors at secondary sites often without the need to enter the vasculature. Within the ascites, ovarian carcinoma cells that have not adhered to the mesothelium can aggregate, forming spheroids.

Besides their spontaneous generation in the ascites of ovarian cancer patients, spheroids can be created *in vitro *from many different cell types for use as three-dimensional tumor models. Research has shown that spheroids tend to be resistant to some chemotherapies and radiation therapy [6-13]. However, few studies have characterized the adhesive or invasive abilities of spheroids, particularly in ovarian cancer, leaving their contribution to dissemination unknown. Despite their undefined role, there is a dearth of studies on cells isolated from ovarian carcinoma effusions. However, previous studies with tumor cells injected into murine models have shown that ascitic tumor cells can penetrate the peritoneal surfaces [14-17]. In humans, it has not been determined whether cells found free-floating in the ascites can be stimulated to become invasive, or if only a few have metastatic potential.

Due to the nature of the multicellular aggregates, it is not feasible to use standard assay techniques to quantitate spheroid cell spreading and invasion. Because patient ascites spheroids range in size, pre-labeling the spheroids either fluorescently or radioactively does not allow differentiation between one very large spheroid versus several smaller spheroids, as both could yield the same output yet give no information about the number of spheroids present or morphologic information such as their change in size. For this reason, we have designed spheroid assays where the spheroids are counted manually and digitally measured at the time of plating and again at each subsequent time-point, to quantitate the actual amount of adhesion, migration, or invasion occurring. These novel assays allow generation of quantitative spheroid data, and are flexible enough to be modified to more closely mimic the peritoneal environment.

Ovarian carcinoma ascites fluid is known to contain a complex mix of secreted factors that can affect the cellular environment of the tumor cells and the mesothelial cells of the peritoneum. Secreted extracellular matrix (ECM) molecules, cytokines, chemokines, proteases, vascular permeability factors, and lysophosphatidic acid have all been detected in the ascites fluid, and many of these molecules have been shown to enhance the growth of tumor cells [18-21]. Considering the unique spread of ovarian carcinoma, the relatively understudied role of the ascites cellular content, and limited knowledge regarding spheroid biology, a more comprehensive understanding of the mechanisms of ovarian carcinoma dissemination is crucial.

In previous studies, we demonstrated that spheroids isolated from ovarian carcinoma patient ascites or created from the NIH:OVCAR5 human ovarian carcinoma cell line can adhere to ECM components and live mesothelial cell monolayers [22,23]. We have also shown that NIH:OVCAR5 spheroids are capable of complete disaggregation on type I collagen, and can invade live mesothelial cell monolayers via β1 integrin interactions and the production of proteases, resulting in foci of invasion 200-fold larger than their initial size within one week [24]. In this study, we continue to address the ability of spheroids to contribute to the spread of ovarian carcinoma by examining the dissemination and invasion of patient ascites spheroid samples *in vitro*.

## Methods

### Materials

Type IV collagen from mouse Engelbreth Holm-Swarm (EHS) tumor was purchased from Trevigen (Gaithersburg, MD). Type I collagen from human placenta was purchased from Southern Biotech (Birmingham, AL). Mouse EHS laminin was purchased from Invitrogen (Carlsbad, CA). Human plasma fibronectin, purified as described, was provided by Dr. James McCarthy, University of Minnesota[25] Human umbilical cord hyaluronan was purchased from Sigma Chemical Co. (St. Louis, MO). Bovine serum albumin was purchased from Pierce Biotechnology (Rockford, IL).

### Antibodies

CD 15 monoclonal antibody (mAb) MMA, CA-125 mAb OV185:1, and CD45 mAb LCA were purchased from Ventana Medical Systems (Tucson, AZ). B72.3 mAb was purchased from Signet Laboratories, Inc. (Dedham, MA). Polyclonal antibodies against CEA and a mAb against Ber-EP4 were purchased from DakoCytomation (Carpinteria, CA). A polyclonal antibody against calretinin was purchased from Zymed Laboratories (South San Francisco, CA).

### Cell culture

The human peritoneal mesothelial cell line LP9 was purchased from the Coriell Cell Repository (Camden, NJ), and maintained in a 1:1 ratio of M199 and MCDB110 media, supplemented with 15% fetal bovine serum (FBS), 2 mM glutamine, 5 ng/ml EGF, 0.4 μg/ml hydrocortisone, and 50 U/ml penicillin/streptomycin. The cells were cultured in 75 mm^2 ^tissue culture flasks in a 5% CO_2 _humidified incubator at 37°C.

### Purification of primary ovarian carcinoma cells

Ascites fluid samples from seven patients diagnosed with stage III or IV ovarian carcinoma, and one patient with primary peritoneal carcinoma (PPC), were obtained through the University of Minnesota Cancer Center Tissue Procurement Facility with approval of the University of Minnesota Institutional Review Board. All patients were newly diagnosed, and had not been treated with chemotherapy prior to our receipt of the samples, as determined by medical records. Ascites tumor cells and spheroids were collected by centrifugation at 100 × g for 10 minutes. Erythrocytes were lysed by resuspending the cells in lysis buffer (10 nM potassium bicarbonate, 155 mM ammonium chloride, 0.1 mM EDTA, pH 7.4) for 5 minutes. The remaining cells were collected by centrifugation at 100 × g for 10 minutes, then layered upon Ficoll-Paque Plus (Pharmacia Biotech, Uppsala, Sweden) and centrifuged again at 400 × g for 15 minutes. The tumor cells were removed from the top of the Ficoll layer and washed in RPMI 1640 media. Aliquots of tumor cells (1 × 10^7 ^cells/ml) were suspended in 10% DMSO and 90% FBS, and stored in liquid nitrogen. The patient sample numbers used in this study correspond to the patient sample numbers assigned in our previous publication [23].

### Immunohistochemistry

Immunohistochemical verification was performed on all ascites samples received. Briefly, paraffin blocks were made from thrombin clots of ovarian carcinoma patient ascites cells following purification. Thrombin clots were prepared by adding 2–3 μl of the ascites cell pellet to 100 μl of human plasma and 50 μl thrombin (Sigma Chemical Co). The thrombin clots were fixed with 10% formaldehyde in PBS, and were paraffin-embedded by the Fairview University Medical Center Pathology Laboratory. 4–5 micron sections were stained with a panel of antibodies against ovarian carcinoma (CA-125), epithelial cells (Ber-Ep4, CD15, B72.3, CEA), mesothelial cells (calretinin), and inflammatory cells (CD45) on an automated immunostainer (Benchmark, Ventana Medical Systems). A pathologist evaluated each sample and verified the presence of 90% tumor cells in all cases.

### Isolation of spheroids

In order to separate the spheroids from the single ovarian cancer tumor cells, an aliquot of ascites tumor cells was defrosted and rinsed with media, then filtered through a 22 μM cell-strainer cap fitted over a 5 ml tube (Becton Dickinson, Franklin Lakes, NJ.) Spheroids retained in the filter were gently rinsed into a collection tube, centrifuged, and resuspended in RPMI media to the desired concentration. We define a spheroid as a cell aggregate of approximately 30–100 μM in diameter. It was usually not possible to visualize one cell from the next in these aggregates, and when sectioned, they were solid throughout (no hollow centers). While the exact number of cells per spheroid varied, they ranged from 10–20 cells up to over one hundred cells in composition.

### Spheroid disaggregation assay

96-well plates were coated overnight at 37°C with 5 μg/ml laminin, fibronectin, type I collagen, type IV collagen, hyaluronan, and BSA in PBS. The wells were then blocked for 1 hour with 2 mg/ml BSA in PBS and then rinsed twice with PBS. A concentration of 5–10 spheroids was added to each of the coated wells. From our previous studies, we determined that 1 hour was an appropriate time for spheroids to begin adhering to an ECM matrix [23,24]. We thus set t = 0 as 1 hour from the initial plating, so that if the plate was not disturbed, the spheroids would not move from their location at the time of plating. Spheroids were digitally photographed at t = 0, incubated at 37°C for 24 hours, and then re-photographed. To track individual spheroids over time, a map of the well was created at t = 0. Reference to the map and the prior time point photograph allowed individual spheroids to be tracked for the duration of the assay. The pixel area of the spheroids at both time points was determined using Adobe Photoshop. The total area included the area of the disaggregated spheroid plus the area of any dispersed single cells in the near vicinity that were most likely to have come from the disaggregated spheroid. Spheroid disaggregation was determined as the fold change in pixel area of the spheroids from 0 to 24 hours. The assay was limited to 24 hours as spheroids did not thrive past 24 hours without sera. Values shown represent the average percent increase in surface area of spheroids from three separate experiments, totalling 20–40 spheroids, ± standard error.

### Spheroid dissemination and invasion assay

To assess invasion, LP9 human mesothelial cells at 35,000 cells/well were added to 96-well plates and allowed to grow to confluence for 96 hours, then were gently rinsed with RPMI. Ascites spheroids were resuspended in complete media to obtain 5–10 spheroids/ml, and 1 ml of the suspension was added to each well atop the mesothelial cell monolayers. Spheroids were digitally photographed 1 hour after plating, and then were incubated at 37°C. The spheroids were photographed again at time-points of 1, 4, and 7 days. Spent media was replaced every three days to prevent the well from becoming too acidic. Two thirds of the media was removed from the top of the well without agitating the mesothelial cell monolayers and was replaced with fresh media. Care was taken to avoid disrupting the wells, and they were inspected carefully before and after media changes to ensure spheroids were not lost. To track individual spheroids over time, a map of the well was created at t = 0. Reference to the map of the well and the prior time point photograph allowed individual spheroids to be tracked for the duration of the assay. Spheroid dissemination was defined as spheroid disaggregation and spreading on top of the mesothelial cell monolayers without forming invasive foci. Spheroid invasion was defined as the establishment of proliferating foci of ovarian cancer cells within the same plane as the mesothelial cell monolayers, such that the mesothelial cell monolayers were pushed aside while the disaggregated spheroid proliferated and grew laterally. Visualization of invasion was achieved by light microscopy without the need for separate labeling of the cells, as the invading ovarian cancer cells were within the same plane of focus as the mesothelial cell monolayers, while cells overlying but not invading the monolayer appeared as a dense cell layer that was slightly out of focus. Both spheroid dissemination and invasion were quantified by calculating the fold change in area, determined by dividing the pixel area of the spheroids at each time point by the spheroid pixel area at t = 0. The data represent the average fold change in area of 50–100 spheroids ± standard error, from a total of four separate experiments for each patient sample.

### Cell proliferation and viability

Ascites spheroid and mesothelial cell monolayer viability and proliferation were determined using a WST-1 assay (Roche Applied Science, Indianapolis, IN.) WST-1 is a tetrazolium salt cleaved to form a formazan dye by mitochondrial dehydrogenase activity. Approximately 20–50 spheroids were plated on ECM components or mesothelial cell monolayers as described above, in 100 μl of media per well. ECM components with media alone, or mesothelial cells alone, respectively, were used as controls. At 0 and 24 hours with the ECM assays, or at 0, 1, 4, and 7 days in the mesothelial cell assays, 10 μl of WST-1 was added to the wells, incubated at 37°C for 3 hours, and the absorbance of each well was read at 440 nm on a SpectraMax Plus^384 ^microplate reader (Molecular Devices, Sunnyvale, CA.) Each experiment was repeated in quadruplicate.

Spheroid and mesothelial cell monolayer viability was also determined using an Annexin V-FITC Apoptosis Detection Kit (BioVision, Mountain View, CA.) Following manufacturer's instructions, cells were labeled with Annexin V-FITC and propidium iodide, and were observed on a Nikon Eclipse E200 microscope using FITC and rhodamine filters (Nikon, Kanagawa, Japan.) Apoptosis appeared as a halo of green at the plasma membrane, with a red stain throughout the nucleus. Cells undergoing apoptosis stained green in the plasma membrane but showed no propidium iodide staining.

### Statistical analysis

The student's t-test was performed as a test of significance with the use of Microsoft Excel 2002 (Microsoft Co., Redmond, WA). P-values of < 0.05 were considered to indicate statistically significant differences.

## Results

### Ascites spheroids disaggregate on ECM components

**Figure 1:**
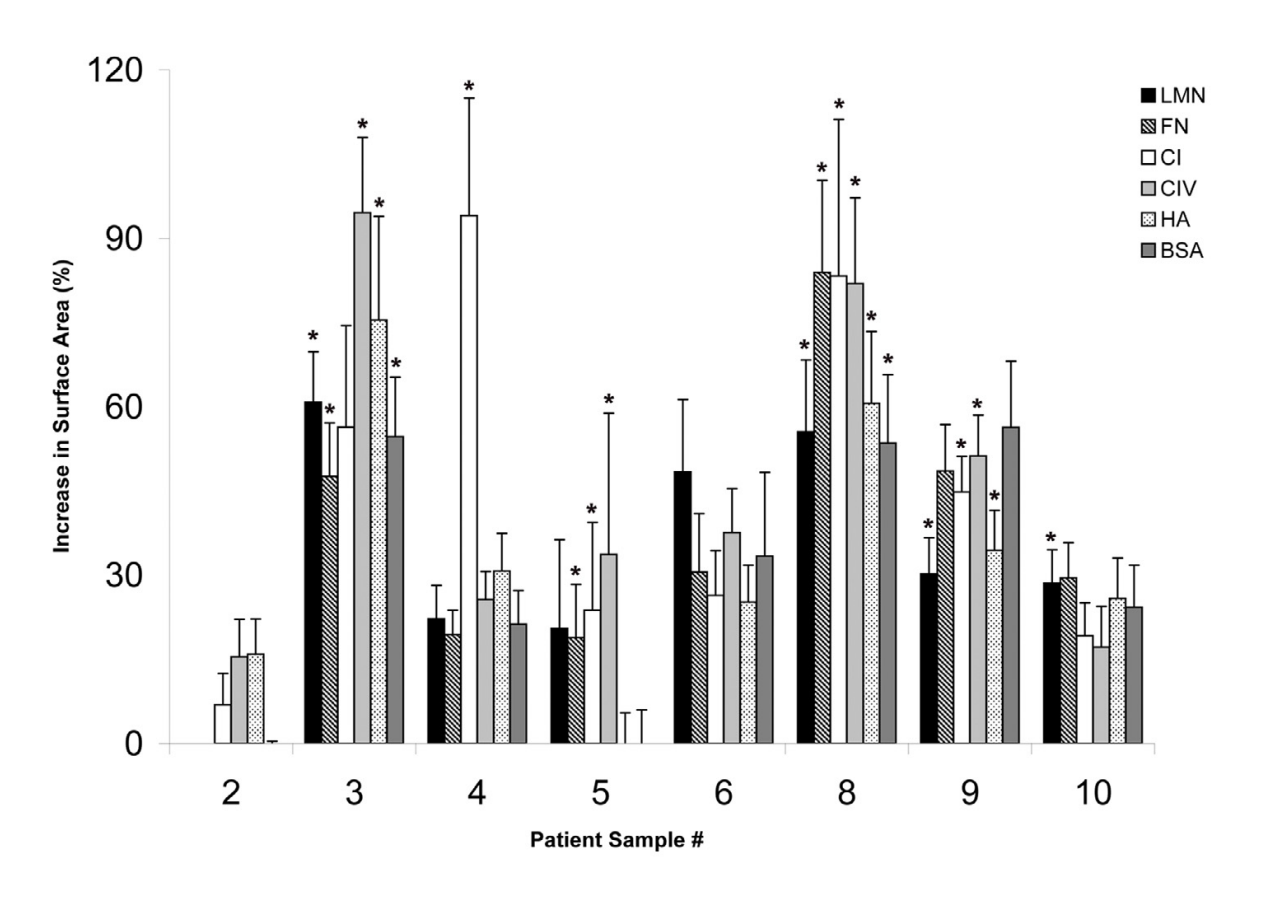
**Disaggregation of ascites spheroids on ECM components**. Ascites spheroids from eight patients were plated on laminin (black bars), fibronectin (striped), type I collagen (white), type IV collagen (light gray), hyaluronan (stippled), or BSA (dark gray) for 24 hours. Values represent the average percent increase in area of spheroids from triplicate experiments, ± standard error; * designates p-values < 0.05.

Spheroids were isolated from the ascites of seven patients with stage III and IV ovarian carcinoma and one patient with primary peritoneal carcinoma. A pathologist evaluated the samples by immunohistochemistry prior to use in assays to verify that each sample matched its diagnosis. Diagnosis, initial CA125 levels, treatment, family cancer history and survival data for the eight patients are included in Table 1. In a previous study from our lab, the adhesivity of these same eight patient ascites samples was tested on various ECM components and live human mesothelial cell monolayers [23]. Here, these ascites spheroid samples were tested for their ability to disaggregate on ECM components. In disaggregation, the spheroids transform from a three-dimensional cell cluster to a flat plaque, as the cells migrate out of the spheroid and dismantle their cell-cell contacts to adhere to and spread on the ECM. 5–10 ascites spheroids were suspended in RPMI and added to each well of a 96-well plate (Fig. 1) coated with 5 μg/ml of laminin (black bars), fibronectin (striped bars), type I collagen (white bars), type IV collagen (light gray bars), hyaluronan (stippled bars), or BSA (dark gray bars) for 24 hours. The spheroids were photographed at the time of plating (t = 0) and again at 24 hours. The fold change in area was calculated by dividing the pixel area of the spheroid at 24 hours by the pixel area at t = 0. Any fold change in area beyond 1 was considered to be significant.

**Table 1:** Patient information.

**Patient #**	2	3	4	5	6	8	9	10
**Diagnosis**	Poorly differentiated papillary serous adenocarcinoma	Papillary serous adenocarcinoma	Primary peritoneal carcinoma with invasive implants	Poorly differentiated serous papillary carcinoma	Serous papillary carcinoma	Serous papillary carcinoma	Metastatic serous adenocarcinoma	Poorly differentiated adenocarcinoma
**Stage**	IIIC	IIIC	IIIC	IIIC	IIIC	IIIC	IIIC	IV
**Grade**	3	2	2	3	3	2	3	3
**Initial CA125**	860	168	1950	2800	366	640	191	6640
**Debulking**	optimal	optimal	optimal	suboptimal		optimal	optimal	optimal
**Treatment**	6 cycles Carbo/Taxol	6 cycles Carbo/Taxol	CDDP/Taxol	Failed several chemos: Taxol, Carbo, Gemzar	Taxol/Carbo 1 cycle	4 drugs/8 cycles	6 cycles (x2) Carbo/Taxol,	6 cycles Carbo/Taxol
**Surgery to Recurrence (Months)**	22	36	8	Progressive disease	Progressive disease	11	18	14
**Overall Survival (Months)**	39	Alive with disease	16	17	1	Alive with disease	Alive	34
**Age at diagnosis**	58	45	63	70	75	29	70	52
**Family History**	Aunt-breast cancer	Mother-ovarian, breast cancer; maternal grandmother-breast cancer; aunt-breast cancer				Mother-ovarian cancer; paternal aunt (x2)-breast cancer; maternal great aunt-gynecological cancer	Sister-breast cancer	Grandmother-uterine cancer; aunt-breast cancer; great aunt (x4)-gynecological cancer; grandmother-ovarian cancer

Patient data was obtained for seven advanced-stage ovarian carcinoma patients and one patient and one patient with primary peritoneal carcinoma. The patient numbers listed correspond with the patient numbers in all subsequent experiments.

**Figure 2:**
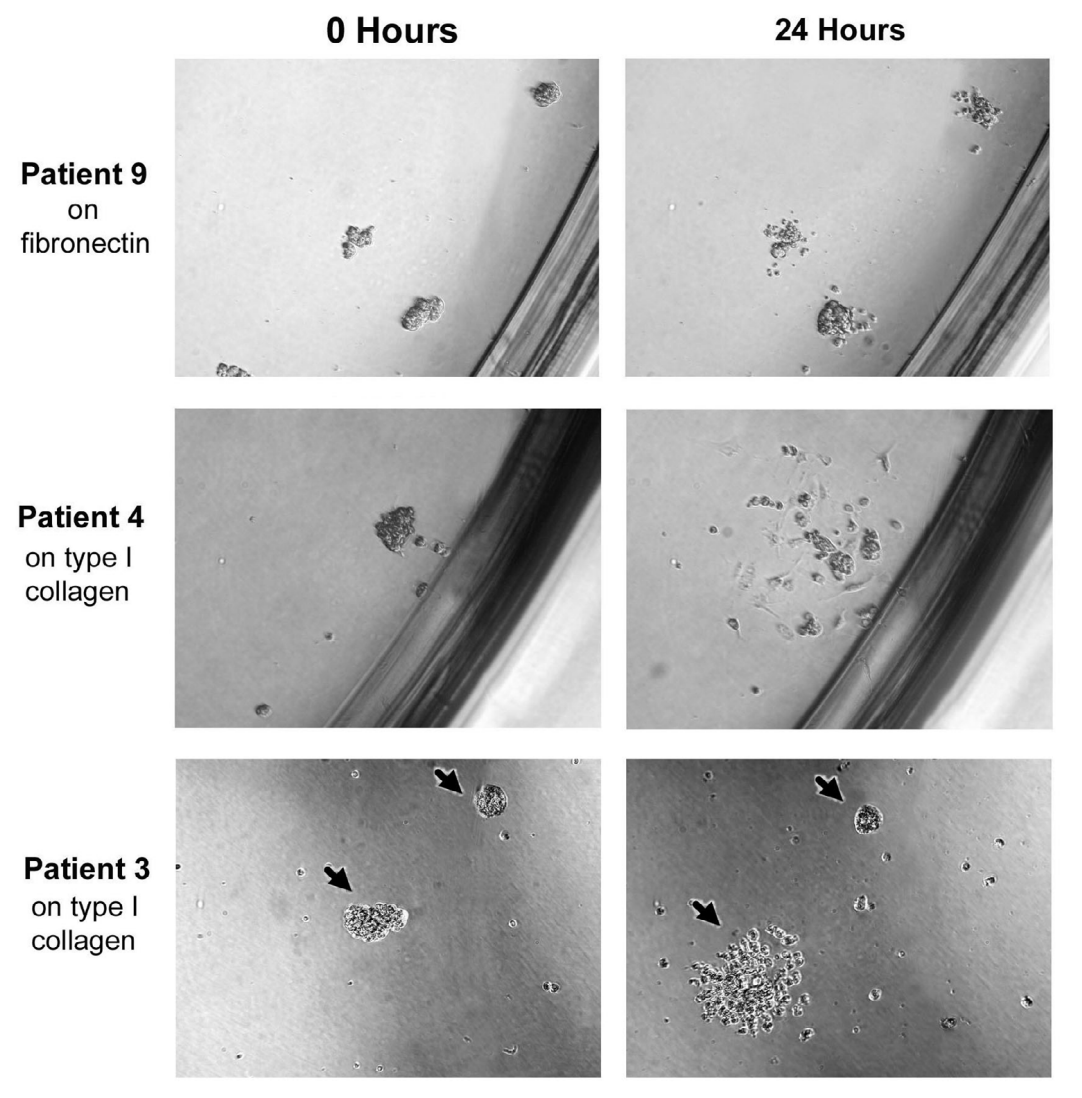
**Ascites spheroids demonstrate variable disaggregation on ECM components**. Patient ascites spheroids were photographed on various ECM components at t = 0 and 24 hours later. Ascites spheroids demonstrated variable migration in response to ECM components. The top panels show outgrowth of patient 9 spheroids on fibronectin, which is representative of the extent of disaggregation for most patient spheroid samples. The middle panels show a spheroid from patient 4 totally disaggregating on type I collagen. The bottom panels show two spheroids, indicated by arrows, from patient 3 on type I collagen, with one spheroid completely disaggregated (left) and one unaltered (right). Magnification = 400×.

In general, ascites spheroids tended to slightly disaggregate on all ECM components, forming a looser spheroid whose cells did not usually flatten or spread to form a monolayer (Fig. 2, top panel). The resulting spheroid surface area after 24 hours typically increased 30–50%, with disaggregation preferentially occurring on type IV collagen and type I collagen. However, individual ascites spheroid samples sometimes demonstrated a greater ability to spread overall, or spread effectively only on specific ECM components. For example, while spheroids from patients 3 and 8 increased in surface area 60% after 24 hours on all ECM components tested, sample 3 preferred type IV collagen and hyaluronan, nearly doubling in size (Fig. 1). Type IV collagen also elicited a near doubling in size from sample 8, as did fibronectin and type I collagen. Samples 6 and 9 both showed about a 40% increase in size, with sample 6 spreading best on laminin, while sample 9 spread similarly on type I collagen, type IV collagen, and fibronectin (Fig. 1). Sample 4, the primary peritoneal carcinoma, exhibited the most dramatic increase in spreading, as it disaggregated extensively on type I collagen more than any other ascites spheroid samples tested (Fig. 1). Samples 2, 5, and 10 showed an average of less than 30% spreading on all ECM components, with few exceptions (Fig. 1).

In some cases, individual spheroids exhibited disaggregation properties that differed from the majority of spheroids in a patient's sample. Thus, even when a majority of a patient's spheroids disaggregated on most ECM molecules, some of the patient's spheroids would not disaggregate or spread. For instance, most of the spheroids from patient 4 demonstrated extensive disaggregation on type I collagen (Fig. 2, middle panel), however some spheroids from this patient did not disaggregate on type I collagen at all. Even within the same well, individual spheroids from the same patient behaved quite differently in response to ECM components. As an example, Figure 2 (bottom panel) shows two spheroids from patient 3 plated on type I collagen. The spheroid on the left completely disaggregated, while the spheroid on the right in the same well did not disaggregate at all. Samples that spread 2-fold or more on any ECM component constituted less than 10% of the total number of spheroids tested for each patient sample.

**Figure 3:**
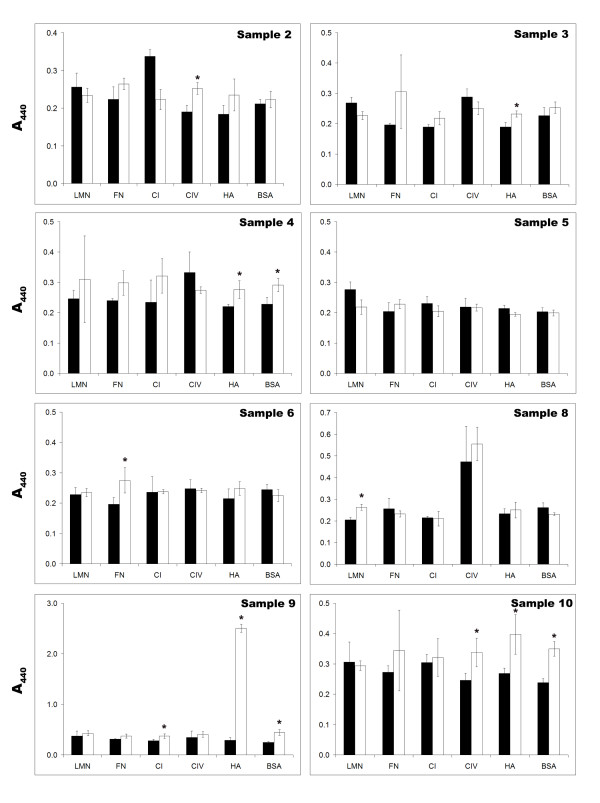
**Ascites spheroids show low levels of proliferation on ECM components in 24 hours**. Patient ascites spheroid samples were added to 96 well plates coated with laminin (LMN), fibronectin (FN), type I collagen (CI), type IV collagen (CIV), hyaluronan (HA), or BSA. At 0 hours (black bars) and 24 hours (white bars), WST-1 was added for 3 hours and then the plates were read on a microplate reader at A_440_. Values represent the average of four wells ± standard deviation; * designates p-values < 0.05.

Spheroid proliferation on ECM components was tested by the cell proliferation agent WST-1 (Fig. 3.) WST-1 readings of ascites samples were compared at 0 and 24 hours, and also to control wells with no spheroids added. Most spheroids did not appreciably proliferate over 24 hours. However, the amount of significant proliferation (p-values < 0.05) seen for individual patient samples sometimes correlated to the ECM components upon which they spread the most. For example, patient samples 2, 6, 8, and 10 showed some proliferation on the ECM components on which they most effectively disaggregated. Sample 3 showed a correlation only on hyaluronan, and sample 9 only on type IV collagen and BSA. In contrast, sample 4 proliferated on fibronectin and hyaluronan even though spheroids from this patient spread extensively on type I collagen. Sample 5 showed no proliferation on any ECM component tested. Taking into account the low levels or lack of proliferation observed, these data suggest that the majority of cells comprising patient ascites spheroids tend to disaggregate and spread rather than proliferate on most ECM components in a period of time limited to 24 hours.

### Ascites spheroids disseminate on mesothelial cell monolayers

**Figure 4:**
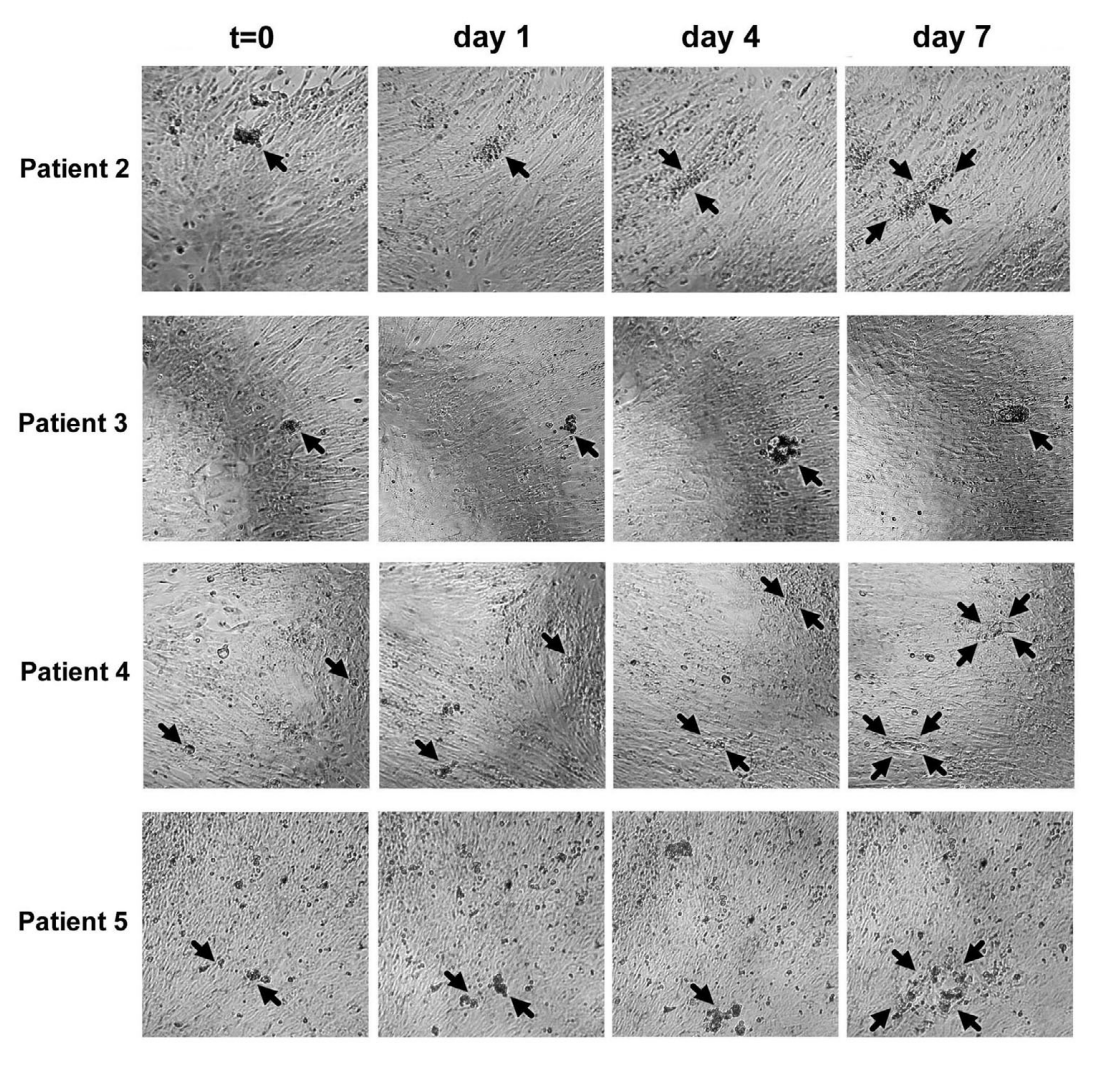
**Patient ascites spheroids #2–5 disaggregate on or invade mesothelial cell monolayers**. Patient ascites spheroids were added to confluent mesothelial monolayers for 7 days to determine their invasive potential. Photos show representative examples of patient samples 2, 3, 4, and 5 at t = 0, and days 1, 4, and 7. Arrows delineate the perimeters of disaggregating or invading spheroids. Magnification = 100×.

**Figure 5:**
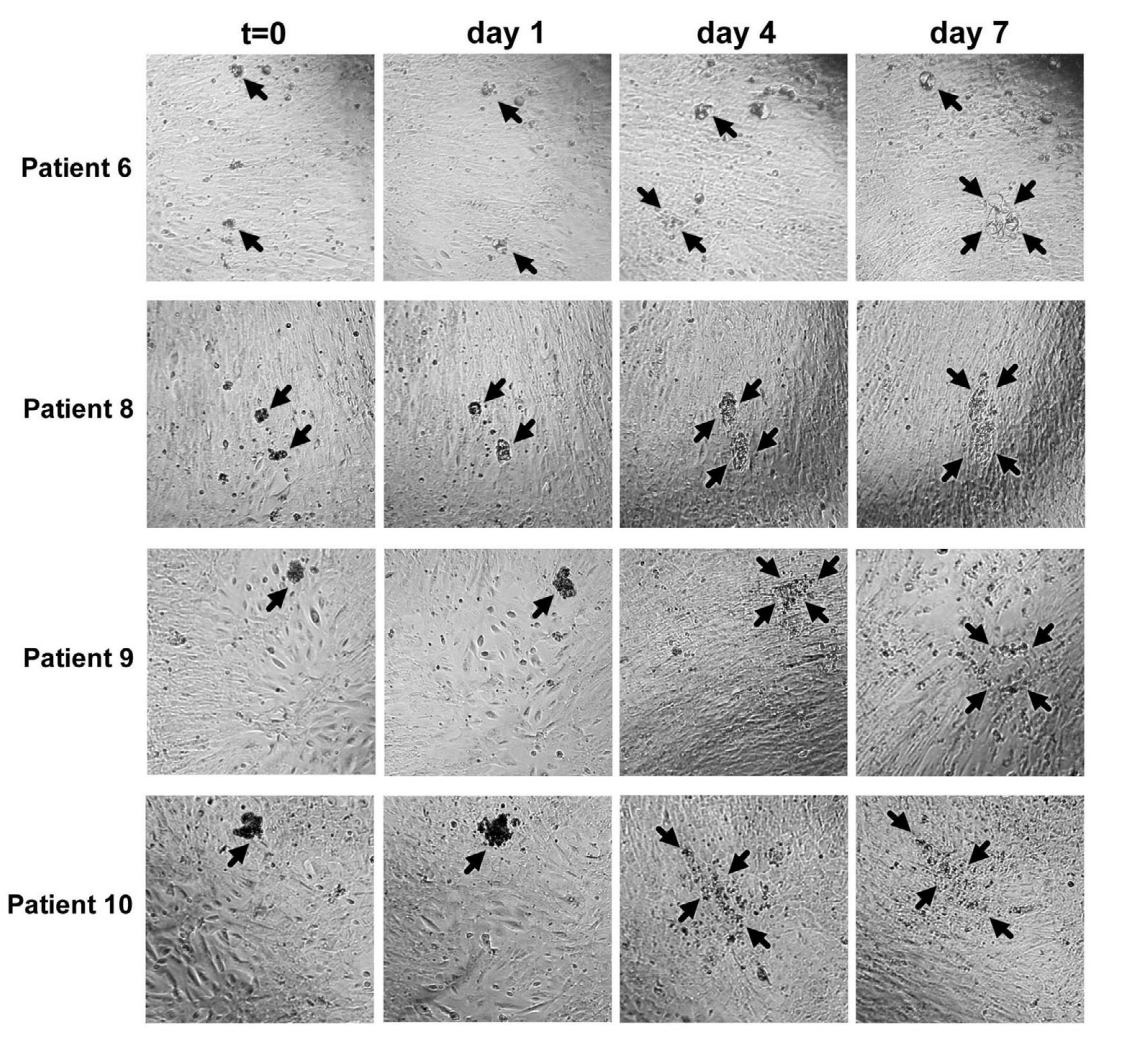
**Patient ascites spheroids #6–10 disaggregate on mesothelial cell monolayers**. Patient ascites spheroids were added to confluent mesothelial monolayers for 7 days to determine their invasive potential. Photos show representative examples of patient samples 6, 8, 9, and 10 at t = 0, and days 1, 4, and 7. Arrows delineate the perimeters of disaggregating spheroids. Magnification = 100×.

The most common site of metastasis in ovarian carcinoma is the peritoneum, which is lined by a monolayer of mesothelial cells. To investigate the ability of ovarian carcinoma spheroids to invade mesothelial cells, invasion assays were performed with ascites spheroids from eight patients. 5–10 ascites spheroids were added to each well of a 96-well plate containing live, confluent human mesothelial cell monolayers, and were incubated for 7 days. The ascites spheroids were photographed at the time of plating (t = 0) and again at days 1, 4, and 7 (Figs. 4 and 5). Disaggregation and invasion was measured as the fold change in pixel area at each time point relative to the pixel area at t = 0.

Frequently, the area of dissemination was visible as a dark discoloration across the top of the monolayer. Upon inspection at a higher magnification, this discoloration was determined to be the dissemination of individual tumor cells from the disaggregated spheroid. In most cases, the areas of dissemination did not significantly increase when assays were extended to 10 days.

**Figure 6:**
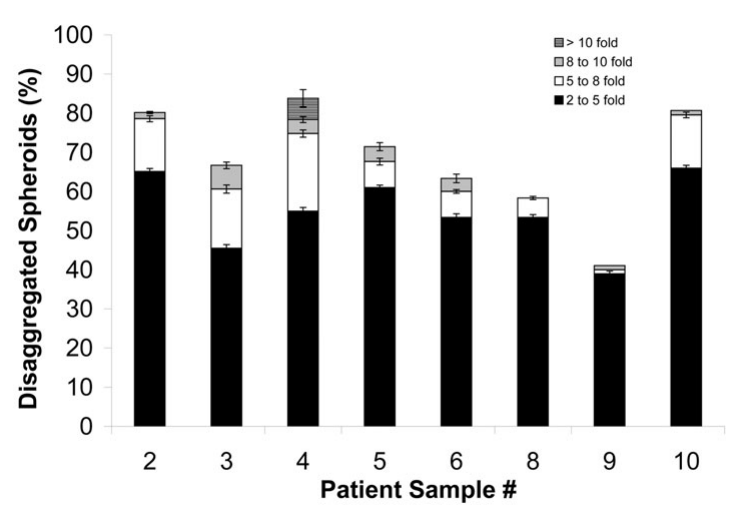
**Ascites spheroids disaggregate on mesothelial cell monolayers**. Ascites spheroids from eight patients were plated on confluent mesothelial cell monolayers for seven days. The fold change in spheroid area was calculated at day 7. Bars represent the percentage of the total number of spheroids that disaggregated 2.0 to 4.9 fold (black), 5.0 to 7.9 fold (white), 8.0 to 10.9 fold (gray), or greater than 11.0 fold (striped) in area over seven days. P-values were < 0.05 for all samples at all time points, with the exception of samples 8 and 10 at day 1.

In general, the ascites spheroids initially increased in size, and then disaggregated on the mesothelial cell monolayers over a period of seven days. Typically, the spheroids did not fully disaggregate in the first 24 hours, although their size increased, suggesting either proliferation or loosening of intercellular contacts. WST-1 assays of ascites spheroid proliferation on mesothelial monolayers, however, were unable to conclusively demonstrate spheroid proliferation (data not shown), suggesting that the enlarged spheroid areas were due to partial disaggregation. By day 4 nearly 50% of the spheroids disaggregated and spread to occupy a surface area that was double their initial size, and had more than tripled in size by day 7 (Figs. 4 and 5). About 50% of the spheroids tested over the course of the assay simply enlarged without disaggregating, demonstrating typically a 2-fold change in size at most. The majority of disseminated spheroids spread between 2 fold and 4.9 fold by day 7 (Fig. 6.) In general, only 5–15% of the spheroids displayed disseminated areas greater than 5 fold. Patient sample 4 showed the greatest amount of dissemination on top of the mesothelial cell monolayers, with more than 30% of the spheroids showing a change greater than 5-fold, and 5% of the spheroids exhibiting changes greater than 11-fold (Fig. 6). In contrast, the majority of ascites spheroids from patients 8 and 9 disaggregated and spread less than 5 fold, with only 2–5% of spheroids spreading more than 5 fold (Fig. 6). Data was statistically significant (p-value < 0.05) for all patient samples at all time points, with the exceptions of patient samples 8 and 10 at day 1.

**Figure 7:**
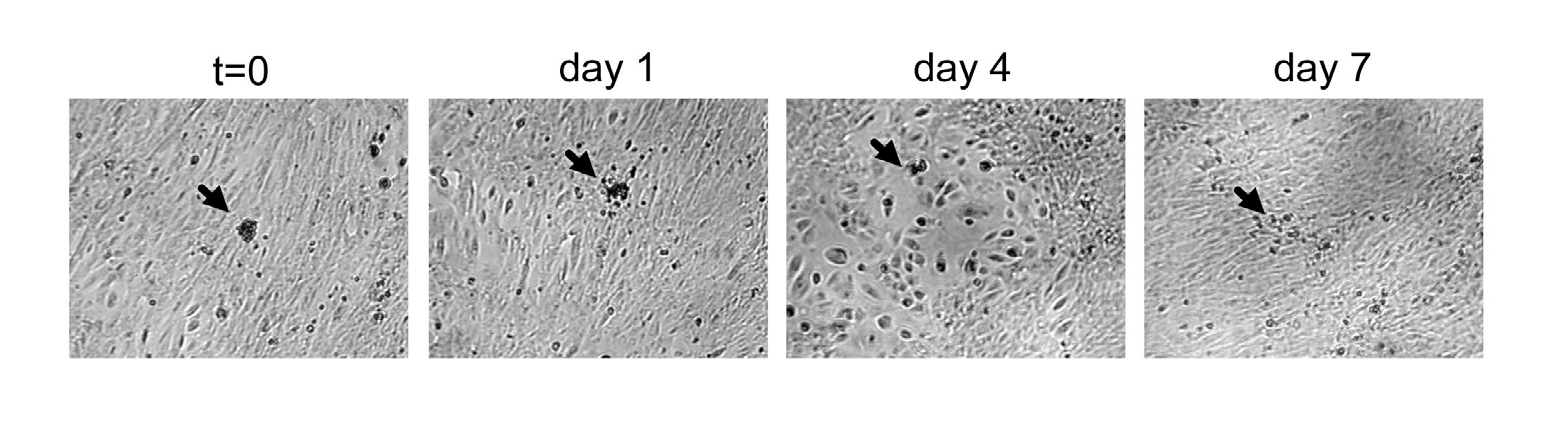
**Ascites spheroids affect the confluence of mesothelial cell monolayers**. (A) The ascites spheroids added to the invasion assays frequently caused areas of sparseness to develop in the area of the mesothelial cell monolayer upon which they were attached. This usually increased up to day 4, but disappeared by day 7. Arrows indicate ascites spheroids. Magnification = 100×.

Previous studies have suggested that the condition of the mesothelium may affect tumor cell invasion [26]. It was often noted during our assays that the morphology of the mesothelial cell monolayer surrounding the area of ascites spheroid attachment altered, growing less confluent and revealing bare patches of tissue culture plastic (Fig. 7). This occurred for about 25% of the spheroids tracked in our assays. However, these sparse areas typically filled in again by day 7, suggestive of mesothelial cell proliferation. Proliferation assays with WST-1 showed that the mesothelial cells, when cultured alone, indeed proliferate up to 7 days, implying that they may be able to repopulate sparse areas during the course of the assay.

**Figure 8:**
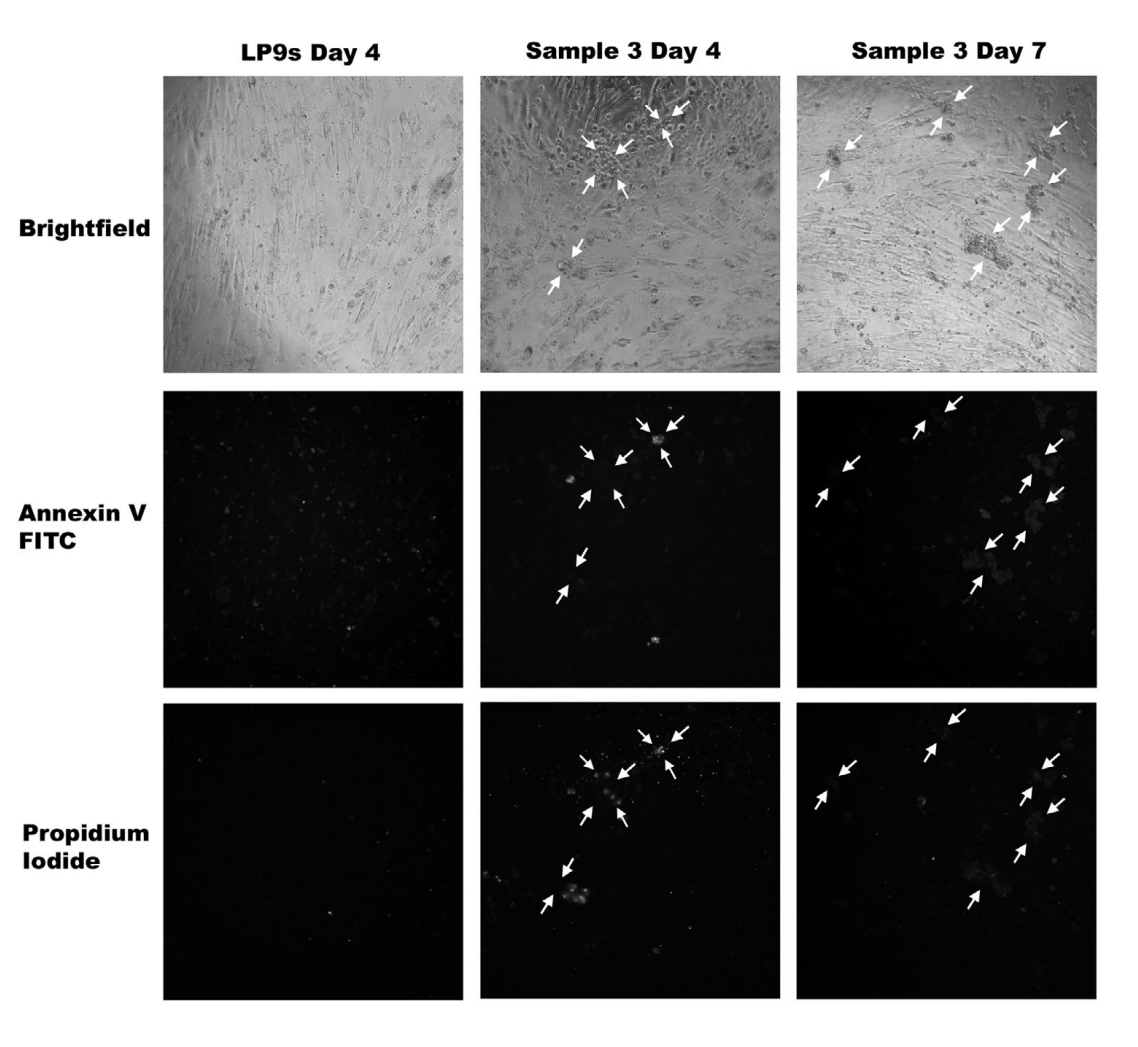
**Ascites spheroids and mesothelial cell monolayers are viable for 7 days**. Ascites spheroids from eight patients were plated on confluent mesothelial cell monolayers in 96-well plates for seven days. At days 0, 1, 4, and 7, wells were stained with Annexin V-FITC and propidium iodide. Panels shows confluent mesothelial monolayers alone (lef column) or with patient ascites sample 3 at day 4 (middle column) or day 7 (right column) with a bright-field image (row 1), Annexin V-FITC staining (row 2), and propidium iodide staining (row 3). Arrows delineate the perimeters of ascites spheroids that have attached and spread on the mesothelial cell monolayer. Magnification = 100×.

To test their viability, ascites spheroids and mesothelial monolayers were stained with an Annexin V-FITC kit (Fig. 8.) Cells undergoing apoptosis stained green with Annexin V-FITC, while cells that had already apoptosed stained red with propidium iodide, and sometimes showed a faint green halo of Annexin V-FITC. In general, the patient ascites spheroids remained alive for the duration of the assays as evidenced by lack of Annexin V and propidium iodide staining, although occasional apoptosis was observed for some individual cells comprising the spheroids. Representative examples from patient sample 3 demonstrate the apoptosis seen in some individual cells of the ascites spheroids and single tumor cells (Fig. 8, middle column) but overall the spheroids remained alive throughout the assay (Fig 8, right column.) Little apoptosis occurred in mesothelial cell monolayers over 7 days (Fig 8, left column.)

**Figure 9:**
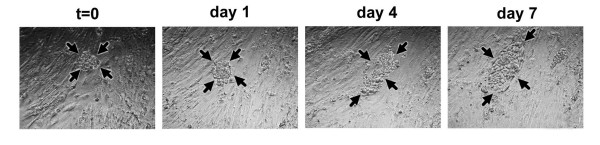
**Patient ascites spheroids are capable of invading a live human mesothelial cell monolayer**. An ascites spheroid from patient 5 is shown invading into a mesothelial cell monolayer. The arrows delineate the area of spheroid invasion and proliferation within the mesothelial cell monolayer. Magnification = 400×.

Despite their ability to attach and spread across the mesothelial cell monolayers, it was rare to see foci of invasion form or extensive proliferation of the ascites spheroid cells. However, we did note ascites spheroids that deviated in behavior. Specifically, about 15% of the ascites spheroids from samples 4 and 5 disaggregated, invaded, and appeared to have proliferated in the mesothelial cell monolayers (patient sample 5 shown in Fig. 9). Invasive ascites spheroids constituted about 5% of the total number of spheroids tested in patient samples, but nevertheless, these data suggest the ascites may contain subclones of spheroids with an invasive potential.

## Discussion

The dissemination of ovarian carcinoma occurs via shedding of cells from the primary tumor with subsequent seeding of the peritoneal cavity, followed by invasion and proliferation at the secondary site. Because widespread peritoneal implantation and advanced disease can often occur without hematogenous spread, determining the invasive potential of the ascites cellular contents is critical.

In earlier studies from our lab, we demonstrated that spheroids created from the NIH:OVCAR5 ovarian carcinoma cell line could adhere to and disaggregate on ECM components, adhere to live mesothelial cells, and extensively invade into live mesothelial cell monolayers [22,24]. Follow-up experiments with ascites spheroids from ovarian carcinoma patients demonstrated that they, too, could adhere to ECM components as well as live mesothelial cell monolayers [23].

In this study, we determined the ability of eight patient ascites spheroid samples to disaggregate on ECM components and invade mesothelial cell monolayers. Patient ascites spheroids typically exhibited some disaggregation, with cells migrating out of the spheroid when placed on a variety of ECM components. The majority of the ascites spheroids increased in surface area on all ECM components tested. In our previous study, NIH:OVCAR5 spheroids completely disaggregated on type I collagen, showing a 6-fold increase in surface area over 24 hours [24]. Here, none of the five ECM components tested induced extensive disaggregation (i.e. more than a 2-fold change in size). Furthermore, the disaggregation seen on ECM components typically was not accompanied by abundant proliferation, although proliferation of particular patient samples sometimes appeared to correlate to the ECM component on which they spread most effectively.

While the levels of patient ascites spheroid disaggregation shown here were not as extensive as the NIH:OVCAR5 spheroids, several possibilities exist to explain this difference. First, NIH:OVCAR5 spheroids were formed *in vitro *over a 48-hour period while ascites spheroids may exist for lengthy periods of time *in vivo*. Thus, patient spheroids may develop extensive cell-cell connections that hinder their ability to disaggregate. Because the ascites spheroid isolation process and subsequent assays require frequent pipetting, filtering, and centrifugation, any spheroids that would disaggregate upon mechanical stimulation are likely to be eliminated from our sample pool, leaving only those spheroids resistant to mechanical disruption and more tightly aggregated. It is possible that the contacts facilitating strong aggregate cohesion may translate to a decreased ability to disaggregate on ECM surfaces. Studies have identified cadherins, desmosomes, tight junctions, gap junctions, and a variety of ECM components including laminin, fibronectin, collagen, and glycosaminoglycans in the cell-cell contacts of spheroids, any of which may hinder their ability to disaggregate upon an extracellular surface [27-31]. Our previous studies have shown that NIH:OVCAR5 spheroids are held together by fibronectin interacting with the α5β1 integrin [22]. Additionally, the patients' spheroids may incorporate molecules such as ECM proteins from the ascites fluid, enabling them to adhere together more tightly or form a matrix that blocks receptors necessary to mediate migration.

Interestingly, some of the spheroids present in patient samples disaggregated quite dramatically on specific ECM components. Ascites spheroids from patient 4 often exhibited complete disaggregation on type I collagen. However, not every spheroid from this patient disaggregated. In fact, spheroids plated in the same well commonly demonstrated differing disaggregation capabilities. This variable response to ECM components suggests that some spheroids within the ascites may develop a metastatic phenotype that attenuates their ability to respond to molecules within their environment. These results may reflect the heterogeneity often seen in solid tumors, where only a few subclones of tumor cells are capable of metastasis.

Previous studies from our lab showed that NIH:OVCAR5 spheroids could invade live monolayers of mesothelial cells, establishing foci of invasion that increased in size up to 200-fold in a week [24]. Here, ascites spheroids from all eight of the patient samples tested were capable of disaggregating on mesothelial cell monolayers or their exposed ECM, typically without actively invading. This is similar to the situation *in vivo*, where tumor cells extensively disseminate throughout the peritoneum without evidence of invading at the time of the first surgery. This suggests that our *in vitro *model accurately mimics *in vivo *conditions. However, some ascites spheroids were able to invade into and proliferate within a mesothelial cell monolayer, also suggesting that a portion of the spheroids in the ascites may constitute an invasive threat.

In those patient samples that demonstrated a more invasive phenotype in our study, not every spheroid was capable of disaggregating and invading. Ascites spheroids plated in the same well did not exhibit the same invasive properties despite sharing an identical environment. In fact, only a few spheroids established foci of invasion. This data implies that the invasive behavior of the ascites spheroids was not merely induced by the spheroids' microenvironment, but likely relied on physiological differences in the spheroids' invasive characteristics. It is widely known that tumors are heterogeneous, and tumor cells can differ greatly in their metastatic potential, such that only a few cells in the primary tumor will become metastatic [32-35]. From the data presented here, it is clear that these same conditions may apply to spheroids in ovarian carcinoma effusions. Further studies where invasive and non-invasive ascitic ovarian carcinoma cells are isolated may allow for the determination of specific characteristics of metastatic cells.

Application of WST-1 to dissemination and invasion assays did not allow a determination to be made regarding spheroid proliferation on mesothelial cells. Based on the design of the dissemination and invasion assay, the confluent mesothelial cells greatly outnumbered the spheroids in each well. When we added an overabundance of spheroids to each well (100–1000 spheroids in an effort to measure spheroid proliferation, we observed a negative impact on mesothelial cell survival. It is possible that only a few spheroids per sample may be significantly proliferative. As only 5% of the total spheroids were capable of invasion, it would be difficult to detect a proliferative spheroid in the WST-1 assay. While the spheroids may not substantially proliferate, they are viable throughout the assay as shown by lack of Annexin V and propidium iodide staining. Some individual cells of the spheroids underwent apoptosis, but the bulk of the spheroids remained alive. When initially recovered from the patients, some of the cells comprising the spheroids were already dead for unknown reasons. It is possible that the one-time freezing and thawing process led to further damage of some cells. However, trypan blue staining of the spheroids during culture revealed that the majority of the cells comprising the spheroids were alive. Thus, while many of the spheroids may not have actively proliferated on the mesothelial cell monolayers, their survival alone still would permit seeding of the mesothelium, which could ultimately result in the development of an invasive subclone.

Of interest, ascites spheroid samples 4 and 5 exhibited the most invasive characteristics *in vitro*, correlating with these patients' shortened overall survival of 16 and 17 months, respectively. It is noteworthy that patient 4, the only sample cultured from a patient with primary peritoneal cancer (PPC), exhibited some of the most invasive characteristics of any spheroids tested. PPC is thought to have a similar biology as ovarian cancer, and the diseases are often considered to be the same. It is tempting to speculate from our evidence here that PPC ascites spheroids may be more invasive than ovarian carcinoma spheroids, although future studies with additional patient samples will be necessary to identify significant differences in the invasiveness of these two cancers.

In our invasion assays, it was sometimes noted that the confluence of the mesothelial cell monolayers was disrupted. This typically occurred in the area surrounding an attached spheroid, leading one to speculate whether factors that the spheroids secrete or induce, such as proteases, may be responsible for this effect. As this effect often disappears by day seven, it is possible that it is a transient effect terminated upon spheroid cell dispersal, allowing the mesothelial cells to proliferate in the damaged area. Indeed, both matrix metalloproteinases and plasminogen activators have been identified in ovarian cancer [36-41]. Intriguingly, similar to our *in vitro *observations, in some ovarian carcinoma patients the peritoneal mesothelium that normally serves as a non-adhesive, protective barrier can become altered, resulting in thickening, vascularity, edema, hyperplasia, and fibrosis [26]. While the cause remains undetermined, a compromised mesothelium may enhance invasion of ovarian carcinoma ascites cells by exposing underlying basement matrices to which the tumor cells can adhere. In support of this, studies have shown that ovarian tumor cells frequently attach to the spaces between mesothelial cells where the underlying ECM is exposed, subsequently leading to mesothelial cell exfoliation and the establishment of invasive foci [42-44]. While one limitation of our study is that it does not show whether invasion is due to mesothelial cell death or exfoliation, our observations with Annexin V staining suggest that the mesothelial cells are alive for the duration of the assay, even when in contact with tumor cells.

## Conclusion

The cellular changes essential for the progression of ovarian carcinoma remain unknown. As little is understood about the ascitic tumor cells and the characteristics necessary to promote their invasive growth, the fact that these cells are often dismissed as non-metastatic is somewhat disquieting. We show here that uncultured ascites spheroid samples from ovarian carcinoma and primary peritoneal carcinoma patients are able to disaggregate on ECM components and human mesothelial cells monolayers, and are sometimes capable of invasion. Based on our data, the biology of the ascites cellular content should be investigated to develop a more comprehensive understanding of the dissemination of ovarian carcinoma.

## Abbreviations

BSA: bovine serum albumin; ECM: extracellular matrix; EHS: Engelbreth-Holm-Swarm; FBS: fetal bovine serum; mAb: monoclonal antibody; PPC: primary peritoneal carcinoma

## Competing interests

The author(s) declare that they have no competing interests.

## Authors' contributions

KB carried out all disaggregation and invasion assays and drafted the manuscript. MB participated in the collection of patient data and clinical analysis of the results. SP analyzed all patient samples by immunohistochemistry and confirmed diagnoses. AS conceived of the study, participated in its design and coordination, and helped to draft the manuscript. All authors read and approved the final manuscript.
